# Exploration of the propagation of transpovirons within Mimiviridae reveals a unique example of commensalism in the viral world

**DOI:** 10.1038/s41396-019-0565-y

**Published:** 2019-12-10

**Authors:** Sandra Jeudy, Lionel Bertaux, Jean-Marie Alempic, Audrey Lartigue, Matthieu Legendre, Lucid Belmudes, Sébastien Santini, Nadège Philippe, Laure Beucher, Emanuele G. Biondi, Sissel Juul, Daniel J. Turner, Yohann Couté, Jean-Michel Claverie, Chantal Abergel

**Affiliations:** 1 Aix-Marseille Université, Centre National de la Recherche Scientifique, Information Génomique & Structurale, Unité Mixte de Recherche 7256; Institut de Microbiologie de la Méditerranée, FR3479, 13288 Marseille, Cedex 9 France; 2 Université Grenoble Alpes, CEA, INSERM, IRIG, BGE, 38000 Grenoble, France; 3 Aix-Marseille Université, Centre National de la Recherche Scientifique, Laboratoire de Chimie Bactérienne, Unité Mixte de Recherche 7283; Institut de Microbiologie de la Méditerranée, FR3479, 13009 Marseille, France; 4 Oxford Nanopore Technologies Ltd, Oxford Science Park, Oxford, UK

**Keywords:** Virology, Cellular microbiology

## Abstract

*Acanthamoeba*-infecting Mimiviridae are giant viruses with dsDNA genome up to 1.5 Mb. They build viral factories in the host cytoplasm in which the nuclear-like virus-encoded functions take place. They are themselves the target of infections by 20-kb-dsDNA virophages, replicating in the giant virus factories and can also be found associated with 7-kb-DNA episomes, dubbed transpovirons. Here we isolated a virophage (Zamilon vitis) and two transpovirons respectively associated to B- and C-clade mimiviruses. We found that the virophage could transfer each transpoviron provided the host viruses were devoid of a resident transpoviron (permissive effect). If not, only the resident transpoviron originally isolated from the corresponding virus was replicated and propagated within the virophage progeny (dominance effect). Although B- and C-clade viruses devoid of transpoviron could replicate each transpoviron, they did it with a lower efficiency across clades, suggesting an ongoing process of adaptive co-evolution. We analysed the proteomes of host viruses and virophage particles in search of proteins involved in this adaptation process. This study also highlights a unique example of intricate commensalism in the viral world, where the transpoviron uses the virophage to propagate and where the Zamilon virophage and the transpoviron depend on the giant virus to replicate, without affecting its infectious cycle.

## Introduction

While for decades most of the focus was given to pathogenic viruses and viruses infecting parasites of human, animals and plants for obvious reasons, they are now recognized as major players in the environment and are by far the most abundant entities in all biotopes including oceans, fresh water, soil [1–5] and are even found in association with multicellular organisms’ microbiotes [6–8]. They have also received a lot of attention with the discovery of *Mimivirus*, the first giant virus with icosahedral capsids visible by light microscopy, enclosing a genome of 1.2 Mb and thousand genes [9, 10]. Many members of the *Mimiviridae* have then been isolated as well as new families such as the proposed Pandoraviridae [11], Molliviridae [12] and Pithoviridae [13], demonstrating that giant viruses now appear ubiquitous in all kinds of environment. Giant viruses infect unicellular eukaryotes, some regulating the populations of bloom forming algae [14–19]. As of today, the *Mimiviridae* family appears composed of several distinct subfamilies [10, 15–18, 20–24] one of which, the proposed Megamimivirinae [5, 17, 18], corresponds to the family members specifically infecting *Acanthamoeba* [20–23]. Members of this subfamilies, collectively refers to as “mimiviruses” throughout this article, can be infected by dsDNA satellite viruses called virophages only able to replicate using the already installed intracytoplasmic viral factory [16, 25–28]. This new type of satellite viruses constitute the Lavidaviridae family [29]. In addition to virophages, mimiviruses can be found associated with a linear plasmid-like 7 kb DNA called a transpoviron [26, 29, 30] making their mobilome uniquely complex among the known large DNA virus families. Sputnik, the first discovered virophage, was found to infect the A-clade Mamavirus [25]. Since it caused the formation of abnormal, less infectious, Mamavirus_A_ particles, it was initially proposed that virophages would in general protect host cells undergoing giant viruses’ infections. Such a protective role was quantitatively demonstrated for the protozoan *Cafeteria roenbergensis*, infected by the CroV [15] virus, in presence of the Mavirus virophage [31, 32]. However, it was later recognized that some virophages replicated without visibly impairing the replication of their associated host virus. This is the case for the mimiviruses of the B or C clades when infected by the Zamilon virophage [28]. On the other hand, members of the A-clade appeared non permissive to Zamilon replication [28]. The resistance to Zamilon infection was linked to a specific locus proposed to encode the viral defence system MIMIVIRE [33–35] but the actual mechanisms governing the virulence of a given virophage vis-à-vis its host virus remain to be elucidated [34, 35]. Transpovirons are commonly detected in *Mimivirus* genomes and might also be involved in the protection of the mimiviruses against the deleterious effects of virophages infection. In this study we took advantage of a newly isolated virophage (Zamilon vitis) and of its ability to propagate different transpovirons to investigate the specificity of the transpoviron/host virus relationship. We also used B- and C-clade strains of mimiviruses originally devoid of transpoviron to investigate the possible role of the transpoviron in the context of virophage co-infections. Finally, we analyzed the proteome of virophage particles replicated on B- and C-clades host viruses, with and without resident transpoviron, to identify transpoviron proteins that could be involved in the co-evolution process allowing the transpovirons to be replicated by mimiviruses.

## Materials and methods

### Viruses isolation

The new giant viruses’ strains were isolated from soil recovered from a French vineyard (43°20′25″N-5°24′51″E, M_C_. vitis), muddy water collected in the Ross river mangrove near Townsville (19°15′42.39″S, 146°49′5.50″E, M_B_. australiensis) and water collected from a well nearby Bamako (12°31′28.6″N 7°51′26.3″W, M_B_. maliensis).

### Virus production

After being treated with antibiotics the different resuspended samples were added to a culture of *Acanthamoeba castellanii* (Douglas) Neff (ATCC 30010^TM^) cells adapted to Fungizone (2.5 μg/ml) and cultures were monitored for cell death as previously described [11–13, 20]. Each virus was then cloned [12] and the viral clones (M_B_. australiensis, M_C_. vitis and M_B_. maliensis) were recovered and amplified prior purification, DNA extraction and cell cycle characterization by electron microscopy (EM). Permissivity to virophage infection on the various giant viruses was analysed using the same protocol to assess the production of virophage after one round of co-infection of each clone with the purified virophage.

### Virus purification

All giant viruses were purified on a CsCl gradient as follows. The cell lysate was centrifuged 10 min at 500 × *g* and the supernatant was centrifuged at 6800 × *g* for 45 min. The viral pellet was washed once with K36 buffer and the viruses were resuspended in CsCl 1.2 density, loaded on a gradual CsCl gradient of 1.3/1.4/1.5 densities and centrifuged at 100,000 × *g* for 20 h. The viral disk was then washed three times with K36 buffer. In contrast to *Sputnik* virophage, Z. vitis and our various giant viruses were still infectious after treatment at 65 °C. We thus could not apply the previously published protocol [26] to separate Z. vitis from the giant viruses. Instead we used several steps of filtration/centrifugation with a final purification on sucrose gradient. The preparation was filtered on 0.2 µm filter and centrifuged at 100,000 × *g* for 90 min. The pellet was resuspended in 5 ml of 40 mM Tris HCl pH 7.5 and filtered on 0.1 µm filter. The filtrate was then centrifuged at 150,000 × g for 1 h, and the pellet resuspended in 0.2 ml of 40 mM Tris HCl pH 7.5 and loaded on a 70%/60%/50%/40% sucrose gradient and centrifuged at 200,000 × g for 24 h. The band corresponding to the virus was recovered with a syringe and washed once with 40 mM Tris HCl pH 7.5. The purification was controlled by negative staining observation using a FEI Tecnai G2 operating at 200 kV (Fig. S1). Competition experiments were performed using a large excess of virophage particles compared with the giant virus (10^3^ for 1).

### Synchronous infections for TEM observations of the infectious cycles

*A. castellanii* adherent cells in 20 ml culture medium were infected with each giant virus with a MOI of 50 for synchronization. After 1 h of infection at 32 °C, cells were washed three times with 30 ml of PPYG to eliminate the excess of viruses. For each infection time (every hour from 1 to 11 h post infection (pi)), 2.5 ml were recovered and we did include them in resin using the osmium-thiocarbohydrazide-osmium method [36]. Ultrathin sections of 90 nm were observed using a FEI Tecnai G2 operating at 200 kV.

### DNA extraction for sequencing

For the M_C_. vitis clone still associated with the Zamilon vitis virophage we did not try to exhaustively separate the giant virions from Z. vitis virions prior to DNA extraction. For each giant virus clone, genomic DNA was extracted from 10^10^ virus using the PureLink^TM^ Genomic DNA mini kit (Invitrogen) according to the manufacturer’s protocol. Finally, for the virophage, after its separation from the giant virus, the DNA was extracted using the same protocol. The purified DNAs were loaded on an agarose gel in search of an extra DNA band suggestive of the presence of an episome that could correspond to a transpoviron. Sequencing of M_C_. vitis and Moumouvirus_B_ australiensis was performed using Illumina technology on an Illumina MiSeq and the Moumouvirus_B_ australiensis transpoviron (matv) sequence transpoviron DNA was extracted and purified from an agarose gel and sent to Nanopore for sequencing. The M_B_. maliensis purified DNA was sent to the Novogene company for library preparation and Illumina PE150 sequencing. Moumouvirus_B_ australiensis genome was sequenced using the Pacbio technology.

### Genome sequencing, assembly and annotation

#### Library preparation for Nanopore technology

DNA was quantified using a Qubit 3.0 Fluorimeter (Thermo Fisher Scientific, MA, USA) following the manufacturer’s protocol, and was found to be 12.7 ng·ml^−1^. 7.5 µl of this DNA was used for library preparation using the RAD002 kit (Oxford Nanopore Technologies, Oxford, UK). Since the input quantity of DNA was lower than recommended for this kit, the active FRM reagent was diluted with three volumes of heat-inactivated FRM, to avoid over-fragmentation of the DNA. The library preparation reaction was set up as follows: the reaction (DNA 7.5 µl, 0.25 × FRM 2.5 µl) was incubated for 1 min at 30 °C followed by 1 min at 75 °C. We added 1 ml of RAD reagent from the RAD002 kit and 0.2 ml of Blunt TA ligase (New England Biolabs, MA, USA) and the reaction was incubated for 5 min at room temperature. The prepared library was then loaded onto a FLO-MIN106 flowcell (version 9.4 nanopores) as per Oxford Nanopore Technologies’ standard protocol.

#### Library preparation for Illumina technology

Genome sequencing was performed using the instrument Illumina MiSeq. Libraries of genomic DNA were prepared using the Nextera DNA Library Preparation Kit as recommended by the manufacturer (Illumina). The sequencing reaction was performed using the MiSeq Reagent Kit v2 (300-cycles), paired-end reads of 150 nt × 2 (Illumina).

The assembly of Moumouvirus_B_ australiensis genome sequence was performed on one SMRT cell of Pacbio data using the HGAP workflow [37] from the SMRT analysis framework version 2.3.0 with default parameters, resulting in 84,565 corrected reads.

The MinION library was sequenced for 1 h on a MinION Mk1B flowcell (Oxford Nanopore Technologies), generating ~220 Mb of sequence data. Basecalling was performed using the 1D Basecalling for FLO-MIN106 450 bps r1.121 [workflow ID: 1200] workflow (Oxford Nanopore Technologies), and yielded 128,623 reads with a mean length of 1701 bases. Data were filtered to remove reads with a quality score below 8, leaving 76,936 reads, and a mean length of 2369 bases. The reads were assembled using Canu with the default parameters, but with the option stopOnReadQuality = false. The matv resulting from this assembly was further polished using the M_B_. australiensis Pacbio error corrected reads.

The assembly of the Megavirus_C_ vitis, Zamilon vitis and Megavirus vitis transpoviron (mvtv) genomes was performed using Spades (version 3.9.0) [38] on MiSeq Illumina paired-end reads and Pacbio long reads when available. Spades was used with the following parameters: careful, *k* = 17, 27, 37, 47, 57, 67, 77, 87, 97, 107, 117, 127, cov-cutoff = off and Pacbio option. For Moumouvirus_B_ maliensis, for which long reads were not available, the assembly was performed using Illumina paired-end reads and Spades (version 3.12.0) with the following parameters: careful, no reads correction, *k* = 33, 55, 77, 99, 127.

Gene annotation of genomic sequences was done using Augustus [39] trained on already published members of the subfamily gene sets. The tRNAs were searched using tRNAscan-SE [40] with default parameters. Functional annotation of predicted protein-coding genes was done using homology-based sequence searches (BlastP against the NR database [41] and search for conserved domains using the Batch CD-Search tool [42]).

### Phylogenetic analyses

The phylogenetic tree of the transpovirons (Fig. 2) was computed using a concatenation of the three conserved genes (orthologous to mvtv_2, mvtv_6 and mvtv_7, see Fig. 2) using the optimal model “LG + G” selected by Prottest [43]. The phylogeny of the giant viruses (including Megavirus_C_ vitis, Moumouvirus_B_ australiensis and Moumouvirus_B_ maliensis, Fig. S2) was performed on a concatenation of the single copy genes shared by all mimiviruses. For that we first predicted the genes using CompareM (https://github.com/dparks1134/CompareM) and clustered them with MCL [44], resulting in 367 conserved genes, and selected among them the 197 present in exactly one copy per virus. Next, the sequences were aligned using Mcoffee [45] and concatenated. Finally, the phylogenetic tree produced from the resulting superalignment was based on the optimal “VT” model selected by Prottest [43]. Average nucleotide identity between the different strains (Fig. S2) was calculated using the OAU tool [46].

### MS-based proteomic analyses

#### Characterization of virion proteomes by data dependent acquisition

For proteomic analysis the virions pellets were resuspended in Tris HCl 40 mM pH 7.5, 60 mM DTT, 2% SDS and incubated 10 min at 95 °C. The protein concentration of the lysates were measured using the 660 nm Protein Assay Reagent appended with Ionic Detergent Compatibility Reagent (Thermo Scientific) and 4 µg of proteins were analyzed as previously described [12]. Two replicates were analyzed per sample, except for Z. vitis purified from M_B_. maliensis and Z. vitis purified from M_C_. chilensis containing matv. Peptides and proteins were identified and quantified using MaxQuant software (version1.6.2.10) [47]. Spectra were searched against the corresponding Megavirus_C_ chilensis, Megavirus_C_ vitis, Moumouvirus_B_ australiensis, Moumouvirus_B_ maliensis, Zamilon vitis, mvtv, matv and *Acanthamoeba castellanii* protein sequence databases and the frequently observed contaminants database embedded in MaxQuant. Minimum peptide length was set to 7 aa. Maximum false discovery rates were set to 0.01 at PSM, peptide and protein levels. Intensity-based absolute quantification (iBAQ) [48] values were calculated from MS intensities of unique + razor peptides. Proteins identified in the reverse and potential contaminants databases as well as proteins only identified by site were discarded from the final list of identified proteins. For each analysis, iBAQ values were normalized by the sum of iBAQ values in the analyzed sample [49]. Only proteins identified with a minimum of two unique + razor peptides in one sample were considered.

### Dominance effect validation by PCR

Cells were grown in T75 flasks and infected either with M_B_. australiensis (carrying matv) at MOI 10 and a large excess (10^3^ for 1 giant virus particle) of virophage carrying mvtv, or with M_C_. vitis (carrying mvtv) at MOI 10 and a large excess of virophage carrying matv. After 40 min of infection, the cells were washed three times and distributed in 12-well plates (1 ml per well). Cells were collected from a well at 45 min, 2 h, 4 h, 6 h, 8 h and 24 h pi. The cells were centrifuged at 1000 × *g* for 3 min, except for the last point at 24 h that was centrifuged at 16,000 × *g* for 10 min. Each pellet was resuspended in 100 µl of PPYG medium and cells were frozen in liquid nitrogen to stop the infection and stored at −80 °C. PCR were performed using the *Terra* PCR Direct *Polymerase* Mix (Clontech) directly on the cell lysates using the following primers:

qPCR-matv_F TCGCTCATTGATTCACTTTGTAC; qPCR-matv_R AATGTATTATGGGCGAATAATGTT; PCR produced an amplicon of 185 bp.

qPCR-mvtv_F GGCATAAGCAGGTTCGAAAT, qPCR-mvtv_R CATGGCGTGATATTGGTGTG; PCR produced an amplicon of 194 bp.

The PCR experiments were stopped after 20 cycles of amplification and 7 µl of the reaction products were deposited on agarose gel (Fig. S3).

### Competition experiments

Cells were grown in T25 flasks (5 ml growth medium) and infected with host viruses carrying one transpoviron at MOI 0.25 and a large excess (10^3^ for 1 giant virus particle) of virophage carrying the complementary transpoviron. After cell lysis, 100 µl of the culture medium containing virophage and host viruses were used to infect another T25 flask containing adherent fresh cells. This process was repeated ten times.

### Selective identification of transpoviron in virions capsids

To distinguish the mvtv and matv transpovirons, two sets of primers were designed:

mvtvP_Fwd_: ACCTTCTTGTGCCTTTACTGC, mvtvP_Rev_: CAGGGTTCGGACGGATTACT; PCR produced a 939 bp amplicon.

matvP_Fwd_: TCGCTCATTGATTCACTTTGTAC, matvP_Rev_: CAAAGGGGAGGAAATAATGGAGA; PCR produced a 263 bp amplicon.

PCR were performed using the *Terra* PCR Direct *Polymerase* Mix (Clontech) directly on the cell lysates after each round of co-infection. To check the stability of the transpovirons over time, up to ten additional rounds of virus production were performed and the presence of the transpoviron was assessed by PCR.

Total DNA was extracted from purified host viruses and virophages using the PureLink Genomic DNA Mini Kit (Thermo Fisher Scientific) and serial dilutions of DNA were performed and deposited on a 0.8% agarose gel ran for 45 min at 100 V. For host viruses, we deposited from 1 to 0.25 µg of total DNA and for virophages, from 1 µg to 62.5 ng DNA. DNA bands were revealed using BET staining and images were recorded on a Chemi-smart 2000WL-20M camera (Fischer Bioblock Scientific).

## Results

### Megavirus_C_ vitis, Moumouvirus_B_ australiensis, Moumouvirus_B_ maliensis and their mobilomes

**Fig. 1:**
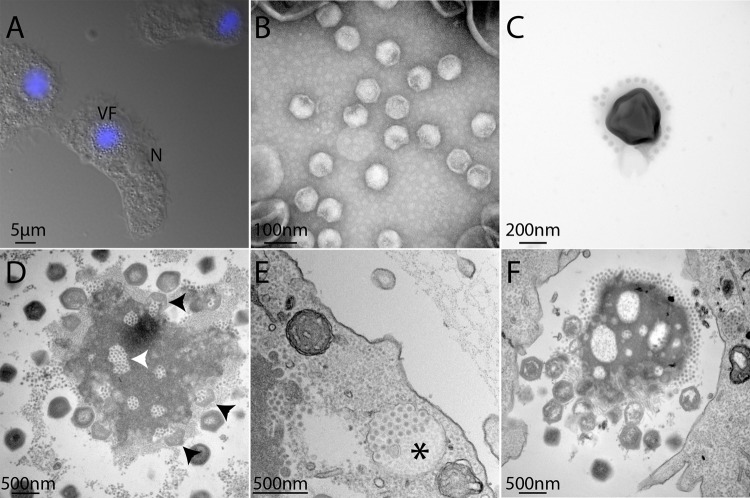
Microscopy images of Megavirus_C_ vitis and its associated virophage Z. vitis. **a** Fluorescence image of DAPI-stained A. castellanii cells infected by M_C_. vitis and its virophage. Viral particles are visible in the periphery of the viral factory (VF). The cell nucleus (N) remains visible but its fluorescence becomes undetectable due to the intense labelling of the VF DNA. **b** Transmission electron microscopy (TEM) of Zamilon vitis particles observed by negative staining electron microscopy; **c** TEM of virophage particles stuck to the giant virus particle (negative staining); **d** ultrathin section TEM of a M_C_. vitis viral factory observed in late infection of *A. castellanii* cells: virophage particles can be seen in holes in the VF (white arrowhead) as well as penetrating a maturing M_C_. vitis particle (black arrowheads); **e** neo-synthesized Z. vitis virophage particles gathered in vacuoles (black star) are seen at the periphery of the infected cell suggesting that they are released by exocytosis. **f** TEM image of an isolated viral factory observed in an ultrathin section of a late infection of *A. castellanii* cells: virophages accumulate at one pole of the VF as well as in holes in the VF while immature and mature M_C_. vitis particles are seen at the opposite pole of the VF (Supplementary movie).

Three samples recovered from various locations (France, Australia and Mali) produced lytic infections phenotypes when added to cultures of *Acanthamoeba castellanii* cells. Viral factories were recognizable in the amoeba cells after DAPI staining and we observed the accumulation of spherical particles visible by light microscopy in the culture medium (Fig. 1a). The corresponding virus populations were cloned and amplified as previously described [12]. In all cases, negative staining EM images confirmed the presence of icosahedral virions of ~450 nm in diameter with a stargate structure at one vertex, as was observed previously for all members of the *Mimiviridae* infecting *Acanthamoeba* cells [20, 28, 50, 51]. For some clones of the giant virus isolated from the French sample, associated icosahedral virions of ~70 nm diameter were also visible, suggesting the presence of virophages (Fig. 1b–f).

Based on its genome sequence, this new isolate was determined to belong to the C clade and named Megavirus_C_ vitis (Fig. S2). Its associated virophage was named Zamilon vitis, in reference to its genomic similarity with the original *Zamilon* virophage [28]. In addition, the genome sequence assembly process revealed the presence of a 7 kb dsDNA sequence homologous to previously described transpovirons [26]. The Megavirus_C_ vitis associated transpoviron was named mvtv. The Australian isolate was found to be a member of Mimiviridae clade B, and was named Moumouvirus_B_ australiensis (Fig. S2). It came with its own transpoviron that we named matv. Finally, the Bamako (Mali) isolate was determined to be another B-clade member, and named Moumouvirus_B_ maliensis (Fig. S2). This last virus was devoid of transpoviron.

For this study, we included the previously isolated Megavirus_C_ chilensis, as a member of Mimiviridae clade C, devoid of transpoviron (Fig. S2). Underscores A, B and C are used throughout this article to indicate the clade origin of the various mimiviruses.

### Comparison of *Acanthamoeba* cells co-infected by B- or C-clade mimiviruses and Z. vitis

The infectious cycles of M_C_. vitis (Fig. S4), M_C_. chilensis, M_B_. australiensis and M_B_. maliensis appeared very similar to that of other mimiviruses, with an initial internalization of the virions in vacuoles, followed by the opening of the stargate and the fusion of the internal membrane with that of the vacuole to deliver the nucleoid in the cytoplasm. After 3 h pi, viral factories develop in the cell cytoplasm, delineated by a mesh of fibres excluding all organelles [20, 52]. Later on, neo-synthesized virions are seen budding and maturing at their periphery. Virophages specifically associated to the mimiviruses are thought to penetrate the cells at the same time as their host viruses, either enclosed in the host virus particles, or sticking to their external glycosylated fibrils (Fig. 1c) [25]. The virophages are devoid of a transcription machinery and thus use the transcription apparatus of the host virus to express their genome once released in the cell cytoplasm [25, 53]. During infection of *Acanthamoeba* cells with M_C_. vitis or M_C_. chilensis in the presence of Z. vitis, regions depleted of electron dense material (“holes”) appeared in the viral factory prior to the assembly of any virion. Z. vitis virophage particles then start to accumulate inside these holes (Figs. 1, S5C, D, Supplementary movie) as early as 4 h pi, before the production of host virus particles. Such infectious cycle is reminiscent of the one described for the association between virophage (Sputnik and Zamilon) and mimiviruses [25, 26, 28], with virophages visible at the periphery of the viral factory, some of them seemingly penetrating inside the maturing giant virions (Fig. 1d). The infectious cycle of Moumouvirus_B_ australiensis during co-infection of *Acanthamoeba* with the Z. vitis virophage was very similar to the one of M_C_. vitis except that instead of holes, the viral factory appeared to segregate the production of virophages in a separate compartment (Fig. S5E, F). In all cases, during the latest stage, virophages were seen in large vacuoles that appeared to migrate toward the cell membrane to be released through exocytosis (Fig. 1e). However, while Sputnik co-infections lead to aberrant and non-infectious *Mimivirus* particles [25, 26], Z. vitis, as other Zamilon virophages, does not visibly impede the replication of its host viruses, abnormal particles of which were never observed [28].

### The newly isolated mimiviruses’ genomes

The dsDNA genome sequence of Megavirus_C_ vitis was assembled into a single contig of 1,242,360 bp with a G + C content of 25%. It was very close to Megavirus Terra1 [54] and to the C-clade prototype Megavirus_C_ chilensis [20] with whom it shared 99.1 and 96.9% identical nucleotides over the entire genome length, respectively (Fig. S2). Moumouvirus_B_ australiensis genome sequence was assembled in one contig of 1,098,002 bp (25% G + C), and that of Moumouvirus_B_ maliensis in one contig of 999,513 bp (25% G + C). As shown in Fig. S2, M_B_. australiensis and M_B_. maliensis belonged to B clade and are closer to each other than to any other moumouviruses, thus initiating a third sub-lineage. Interestingly, the B clade appeared the most divergent among mimiviruses.

### Zamilon vitis genome

In addition, we determined the 17,454 bp genome sequence (30% G + C) of Zamilon vitis. It was closely related to that of other virophages infecting the B and C clades, sharing 97.8% identical nucleotides with the prototype *Zamilon* virophage [28]. The 20 predicted proteins were all conserved in other virophages infecting mimiviruses, sharing 40–80% identical residues with Sputnik [25], their most distant homologue.

### The mvtv and matv transpovirons

**Fig. 2:**
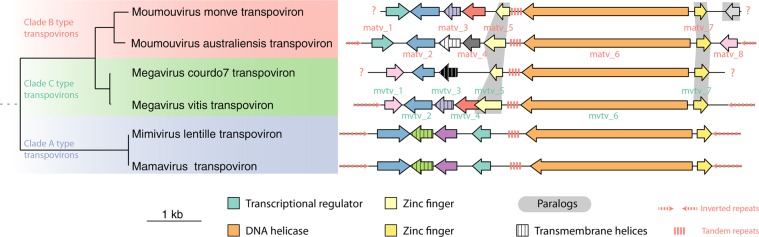
Phylogeny and genomic organization of transpoviron sequences. The phylogenetic tree (on the left) was computed from the concatenated sequences of shared orthologous predicted proteins using PhyML [45] with the LG + G model. Bootstrap values (not shown) are all equal to one. The genomic organization (right) shows orthologous genes represented with identical colours and paralogous genes (in a given genome) are highlighted in grey. Gene names are indicated for matv and mvtv.

Finally, we determined the genome sequences of the two new transpovirons. The mvtv DNA sequence was 7417 bp (22% G + C) in length and closely related to the one associated with Megavirus_C_ courdo7 (98% identical nucleotides). The matv DNA sequence was 7584 bp in length (22% G + C) and was related to the one associated with Moumouvirus_B_ monve (89% identical nucleotides). The reconstructed phylogeny of all the known transpoviron genomes clearly showed that they fell into three distinct clusters, mirroring the tripartite clades structure of the host viruses from which they were isolated (Fig. 2).

Transpovirons exhibited terminal inverted repeats (TIRs) of 520 nt for Mamavirus_A_ and Lentille virus_A_ transpovirons (lvtv), 380 nt for matv and 510 nt for mvtv. TIRs were missing from the transpovirons associated to Moumouvirus_B_ monve and Megavirus_C_ courdo7, most likely because their published sequences are incomplete [26]. TIRs are well conserved within clades (90% of identical nucleotides between Mamavirus_A_ transpoviron and lvtv) but diverged between clades (56% identical nucleotides between lvtv/mvtv and 53% between mvtv/matv). A tandem repeat (TR) of 180–600 nt was present in the centre of all sequenced transpovirons, in an intergenic region 3′ from a conserved helicase (Fig. 2). These TRs were also well conserved within clades (80% identical nucleotides) and divergent between clades (39% identical nucleotides). It is worth mentioning that an evolutionary link between virophages and transpovirons has been proposed [55]. Three predicted proteins were found in all transpovirons (i.e. core genes): a helicase (Mvtv_6/Matv_6), a protein of unknown function (Mvtv_2/Matv_2) and a zinc-finger domain-containing protein (Mvtv_7/Matv_7), (Fig. 2). In addition, all transpovirons encode a small protein with a central transmembrane segment as their only recognizable similarity (Mvtv_3/Matv_3). Seven predicted proteins were shared by at least two transpovirons, including a transcriptional regulator conserved in clades A and B (Matv_1), a protein paralogous to the above core zinc-finger-domain-containing protein and conserved in clades B and C (Mvtv_5/Matv_5), and five other predicted proteins without any functional signature. Finally, four proteins of unknown function were unique and had no detectable homologue in the other transpovirons.

We analyzed the proteome of M_C_. vitis virions in search of transpoviron proteins specifically associated to this host virus. We identified three transpoviron proteins, Mvtv_3, a putative membrane protein that could be anchored in the giant virus membrane, and Mvtv_2 and Mvtv_4, two putative DNA-binding proteins (Fig. S6, Table S1).

Given that all known virophages infecting mimiviruses have been isolated in the presence of a transpoviron, we also expected the presence of transpoviron-encoded proteins in Z. vitis virions. We thus analyzed the protein composition of the purified Z. vitis particles produced with M_C_. vitis. The proteomic study confirmed this prediction but suggests a specific interaction between the transpoviron proteins and the virophage in one hand, and the transpoviron proteins and the host virus particles in the other hand. Indeed, in virophage particles, we consistently identified two transpoviron proteins, Mvtv_7, a putative DNA-binding protein and in lesser amount the predicted helicase Mvtv_6 never identified in the M_C_ vitis particles in the absence of virophage particles (Table S1). Four additional proteins were also detected but in much smaller amount, three predicted DNA-binding protein (Mvtv_5, Mvtv_2 and Mvtv_4 in decreasing amounts) and a protein with unknown function, Mvtv_1. These four proteins were also seen in the total proteome of the M_C_. vitis + Z. vitis virions. In contrast, they were all absent from the proteome of the cloned M_C_. Vitis particles (Fig. S6). Thus, the transpoviron encodes different subsets of proteins that might be specifically involved in their packaging in two alternative vehicles: the virophage or the host virus particle.

### Clade specificity of transpovirons

First, we verified that Z. vitis virophage replication was restricted to host viruses from the B and C clades, as previously described for Zamilon virophages (Table 1) [28]. We also verified by PCR that the M_C_. vitis clone cleared from virophage and replicated on *A. castellanii* cells remained associated with its transpoviron mvtv.

**Table 1:** Permissivity of the host Megavirinae to Z. vitis virophage and their selectivity for the transpovirons.

Clade	Giant virus	Permissivity to Z. vitis	Selectivity for transpoviron	
			Z. vitis + mvtv	Z. vitis + matv
**A**	*Mimivirus*	−	mvtv−	matv−
**C**	M_C_. chilensis	+	**mvtv+**	matv+
	M_C_. chilensis + **mvtv**	+	**mvtv+**	**mvtv+** matv+
	M_C_. chilensis + matv	+	matv+ **mvtv+**	matv+
**C**	M_C_. vitis + mvtv	+	**mvtv+**	**mvtv+**
**B**	M_B_. australiensis + matv	+	**matv+**	**matv+**
**B**	M_B_. maliensis	+	mvtv+	**matv+**
	M_B_. maliensis + **matv**	+	**matv+** mvtv+	**matv+**
	M_B_. maliensis + mvtv	+	mvtv+	mvtv+ **matv+**

Transpovirons originally associated to a given host virus are underlined. Most abundant transpovirons in host particles are shown in bold

**Fig. 3:**
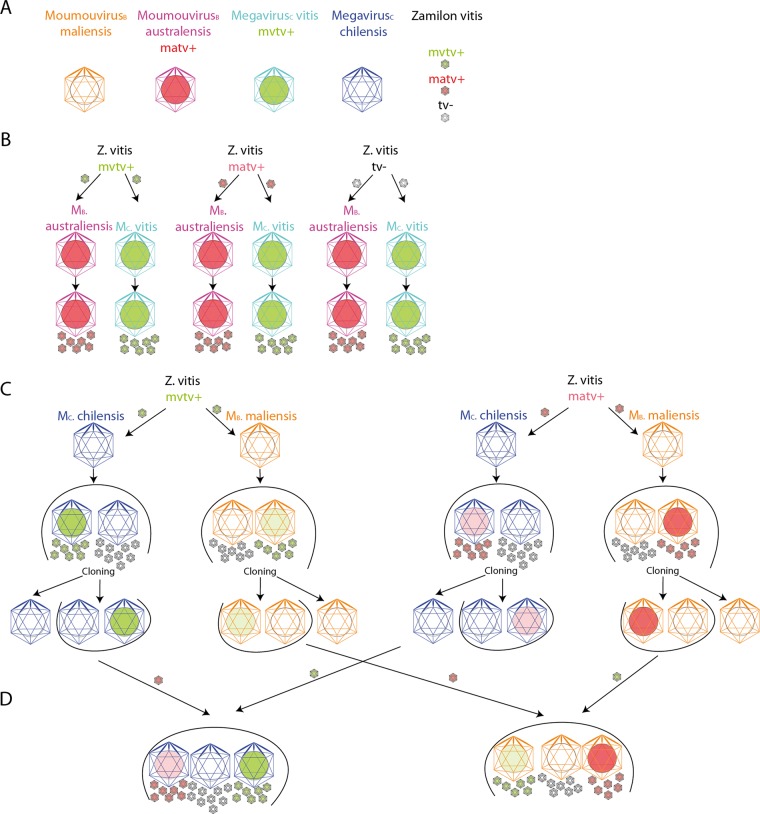
Dominance effect versus permissive effect. **a** Viruses used in this study. M_B_. maliensis is represented in orange, M_B_. australiensis in purple, M_C_. vitis in cyan and M_C_. chilensis in dark blue. The transpovirons are represented as coloured circles (green for mvtv and pink for matv) inside the giant virus and virophage capsids. **b** Dominance effect of the resident transpoviron (mvtv in M_C_. vitis, green circle in cyan capsid; matv in M_B_. australiensis, pink circle in purple capsid) over the one carried by Z. vitis. Empty Z. vitis (black contour, white capsid) do acquire the resident transpoviron upon replication; **c** Permissive effect: the two type of transpovirons can be imported and replicated by empty M_C_. chilensis (dark blue capsid, white circle) and M_B_. maliensis (orange capsids, white circle), although with different efficiencies. **d** Combination of the dominance and permissive effect. Colour intensities of the circles (pink for matv, green for mvtv) illustrate the abundance of the transpovirons in the host and virophage particles.

Purified virophage virions carrying the mvtv transpoviron were then used to co-infect *A. castellanii* with two C-clade megaviruses (M_C_. vitis/mvtv and M_C_. chilensis w/o transpoviron) and two B-clade moumouviruses (M_B_. australiensis/matv and M_B_. maliensis w/o transpoviron) to assess whether the transpovirons were specific to a given clade of mimiviruses (Fig. 3a).

We performed specific PCR for each transpoviron after each round of co-infection and for each additional round of production (up to ten) to assess the presence of matv and mvtv DNA in the cultures after cell lysis. We also performed a proteomic analysis of the resulting virophages to assess the presence of transpoviron proteins (Table S1).

Co-infection of the virophage (carrying mvtv) with M_B_. australiensis (carrying matv) surprisingly produced a unique population of neo-synthesized virophage particles carrying the matv transpovirons (Table 1, lane 6). The proteomic analysis of the purified virophage capsids also evidenced the replacement of the mvtv proteins by their orthologues in matv, matv_7 and matv_6 and to a lesser extend matv_2. In contrast the orthologues of Mvtv_1 (Matv_8) and Mvtv_5 (Matv_5) were not detected (Table S1). Moreover, PCR performed along the infection cycle of M_B_. australiensis (carrying matv) and the virophage (carrying mvtv) did not show an increase in mvtv while the matv genome was clearly replicated (Fig. S3A). These results suggested that the host virus strongly favour the replication of its natively associated transpoviron (Table 1, Fig. 3b). If a different one is brought in by the virophage, it is lost and replaced by the one replicated by the host virus, a result we refer to as the “dominance effect”. Consequently, two populations of virophages carrying either mvtv or matv were at our disposal. We confirmed the dominance effect by co-infection of M_C_. vitis carrying mvtv with virophages carrying matv (Table 1, lane 5). Again, we observed the replacement in the virophage particles of matv (DNA and proteins) by mvtv (DNA and proteins). We also confirmed that the mvtv genome was actively replicated while the amount of matv genome remained stable along the infectious cycle (Fig. S3B). We then used the virophages carrying either mvtv or matv to infect transpoviron-free B-clade (M_B_. maliensis) and C-clade (M_C_. chilensis) host viruses. We found that the virophage succeeded in transmitting each transpoviron to each “empty” B- or C-clade host viruses (Table 1, lanes 2 and 7), a result we refer to as the “permissive effect”. However, we observed that matv was preferentially replicated by M_B_. maliensis and mvtv by M_C_. chilensis (Table 1). By cloning we showed that the resulting populations of B- and C-clade host viruses were mixtures of transpoviron positive and transpoviron negative particles (Fig. S7). Furthermore, virophage particles produced by transpoviron negatives clones were also devoid of transpoviron (DNA and proteins), indicating that although transpovirons can be carried by virophages, their replication is performed and controlled by the host virus.

We finally took advantage of the permissive effect to produce two populations of clade B (M_B_. maliensis) and C (M_C_. chilensis) host viruses, each carrying the matv or mvtv transpoviron. We then challenged them using virophages carrying the other transpoviron. We performed PCR specific for either transpoviron after each round of virophage co-infection (up to ten successive rounds of virophage production) and assessed their presence after cell lysis.

The proteome of the virophages particles was also analysed in order to assess the presence of transpoviron proteins possibly associated to the transpoviron DNA. The results of the various experiments are presented in Table 1 and Table S1 and are interpreted in Fig. 3.

When we infected populations of M_C_. chilensis carrying matv or mvtv with virophages carrying the complementary transpoviron, the resulting population of M_C_. chilensis and virophages became positive for the two transpovirons (Table 1, lanes 3 and 4). This apparently violated the strict “dominance effect” observed with M_C_. vitis and M_B_. australiensis. However, the persistence of a subpopulation of empty M_C_. chilensis virion (i.e. devoid of transpoviron) might also explain our results without refuting the dominance rule. In that case, the replication and propagation of the competing virophage-borne transpoviron would be performed by the transpoviron-null (empty) M_C_. chilensis subpopulation. We investigated this possibility by cloning virions from the mixed mvtv/matv M_C_. chilensis population and examining their transpoviron content. Strikingly, we never observed M_C_. chilensis virions simultaneously carrying both types of transpovirons. On the other hand, we observed either mvtv positive (mvtv+) or matv positive (matv+) M_C_. chilensis clones, as well as others devoid of transpovirons (Fig. 3). This result also suggests that the association between the host virus and the transpovirons are not stable when resulting from a first encounter. In the case of M_B_. maliensis in the presence of virophage matv, the replacement of the mvtv transpoviron from host particles appeared to be faster, which resulted in the rapid disappearance of mvtv after only six rounds of replication. We also observed that the loss of the transpoviron over host virus replication, in the absence of virophage, was more rapid for M_B_. maliensis than M_C_. chilensis suggesting the association between the host virus and the transpoviron was not stable. The cloning step provided virophage-free host virus clones with which to replicate these competition experiments: the matv+ or mvtv+ clones infected by virophages carrying the complementary transpoviron again produced a mixed population of matv+, mvtv+ and transpoviron-null virions (Table 1, lanes 8 and 9). Thus, the persistence of particles devoid of transpoviron allows us to conclude that our results are a combination of the “dominance effect” applied to the subpopulation of transpoviron positive virions and of the “permissive effect” applied to the transpoviron-null subpopulation. In the resulting virophage particles, the only transpoviron proteins consistently identified were Mvtv_7/Matv_7 and Mvtv_6/Matv_6. As expected, they were absent from virophages devoid of transpoviron (Table S1).

To elucidate whether the transpoviron could have a protective role against infection of the host virus by the virophage, we compared the infectious cycles of *Acanthamoeba* cells infected by M_C_. chilensis carrying matv, mvtv or without transpoviron. They were strikingly similar both in terms of cycle length and virus production yields. Transpovirons are thus not key, at least in laboratory conditions, in regulating the permissivity of mimiviruses to virophage infection.

## Discussion

Mimiviruses are unique in their association with two distinct (often co-existing) dependent entities, virophages and transpovirons, somewhat reminiscent of phages and plasmids afflicting bacteria. As for the virophage, the presence of host virus-like regulatory elements (terminator hairpin and late promoter [56–59]) flanking the transpoviron genes suggest that they also use the host virus transcription machinery rather than that of the cell. The transpoviron might also rely on the host virus DNA replication machinery, in absence of transpoviron-encoded DNA polymerase. Our competition experiments between the mvtv *vs*. matv transpovirons resulted in the replication of only one transpoviron. Interestingly, the “winner” corresponds to the type originally associated to the host virus (mvtv for M_C_. vitis and matv for M_B_. australiensis, Table 1), a phenomenon we called the “dominance effect”. This finding was also confirmed by the immediate replacement of mvtv by matv proteins in virophage particles synthetized with M_B_. australiensis. However, this result is not simply due to a strict clade-wise specificity. The use of transpoviron-free host virus particles allowed us to demonstrate that M_C_. chilensis and M_B_. maliensis can replicate and incorporate each transpoviron, independently (Table 1). Yet, we observed a marked difference in permissivity, with B- and C-clade host viruses favouring their cognate transpoviron types. The central TRs sequences and the TIR flanking the transpovirons replicated by A-clade vs. B- or C-clade host viruses are markedly different. These differences might cause the lack of replication of matv and mvtv in the A-clade *Mimivirus*. The lesser differences between the B- and C-clade transpovirons might then explain why both of them can still be replicated by M_B_. maliensis and M_C_. chilensis. In these host viruses, the competition experiment resulted in the simultaneous replication of both transpovirons. However, the sub-cloning of the mvtv+/matv+ population resulted in mvtv+ only, matv+ only, or transpoviron negative clones. It also appears that neither of the two transpovirons remains stably associated with a host virus for which it was a first encounter, while a preference could emerge once a stable association has been established by co-evolution (i.e. M_B_. australiensis with matv, M_C_. vitis with mvtv). Finally, in virophage particles there were fewer copies of matv either produced with M_B_. australiensis, M_B_. maliensis or M_C_. chilensis and fewer copies of mvtv produced with M_C_. chilensis or M_B_. maliensis, compared with the number of copies of mvtv produced with M_C_. vitis. Different transpovirons, even efficiently replicated by the host virus, thus appear to be loaded at different efficiencies in the virophage particles (Fig. S8). A similar result was previously described for the Lentille virus transpoviron that could only be detected in Sputnik2 virophage particles using FISH experiments [26]. A consequence of such suboptimal associations was the production of virophages devoid of transpovirons that could then be used to identify candidate proteins involved in the transpoviron/virophage association. The only difference between virophage particles carrying or not transpovirons was the recurrent presence of two transpoviron-encoded proteins (Mvtv_7/Matv_7 and Mvtv_6/Matv_6) together with the DNA molecule as an episome (Table S1, Fig. S8) suggesting the virophage was a mere vehicle for the transpoviron. These proteins are conserved in all transpovirons, are predicted to be DNA-binding, and were not identified in the proteome of the host virus. Instead, the most abundant transpoviron proteins in M_C_. vitis virions were two predicted DNA-binding proteins (Mvtv_2 and Mvtv_4) and one predicted membrane protein (Mvtv_3) that could be anchored in the host virus membrane (Table S1). All transpovirons encode a short predicted membrane protein although their primary sequence does appear to be conserved. The dominance effect is reminiscent of plasmids incompatibility or entry exclusion for related plasmids [60–63] or superinfection immunity [64] and superinfection exclusion [65] used by prophages to prevent superinfection by other bacteriophages. For the latter, DNA injection is often inhibited by a membrane-bound protein [66–68], paralleling the eventual role of the transpoviron-encoded membrane protein in giant virus particles carrying a transpoviron.

Since the transpoviron genome is present in both the host virus and the virophage particles, the transpoviron DNA might also adopt a different organization depending on the vehicle (host virus or virophage particles) used for its propagation. The Mvtv_7/Matv_7 and Mvtv_6/Matv_6 proteins could be involved in the packaging or delivery of the transpoviron in and from the virophage particle, while the Mvtv_2/Matv_2 and Mvtv_4 proteins could play a similar role vis-à-vis the host virus and the Mvtv_3 membrane protein may play a role in the dominance effect. Further studies are needed to elucidate the mechanisms at work in packaging and delivery of transpoviron genomes as well as in transpoviron dominance.

The first two types of virophage that have been discovered, Sputnik and Mavirus, respectively infecting *Mimivirus* [25] and *Cafeteria roenbergensis* [31] are strongly deleterious to their host viruses, diminishing the production of infectious particles [25, 26, 69] or stopping it altogether [31, 32], effectively protecting the cellular hosts. As parasite of another parasite (the giant virus), these virophages are bona fide hyperparasites [70, 71]. The detection of many additional virophage-related sequences in aquatic environment together with that of *Mimivirus*-like viruses suggested that they might have a significant ecological role in regulating the population of the giant viruses and of their cellular host (micro-algae or heterotrophic protozoans) [72–74]. However, the hypovirulence (of the host virus) induced by the hyperparasite may ultimately limit its own reproductive success [70]. Thus the evolutionary trajectory of the virophage/host virus/cellular host parasitic cascade may remain antagonistic or end up in a mutualistic or commensal relationship. The uniquely complex tripartite parasitic cascade transpoviron/virophage/host virus analyzed in this work, where none of the actors appears to have a detrimental effect on the others, at least in laboratory conditions, might be at a neutral equilibrium reached as a stable compromise after eons of intricate antagonistic evolution. To the best of our knowledge, the relationship between the transpoviron, the Zamilon virophage and their host giant virus analysed in this work represents the first example of bipartite commensalism in the viral world.

## Supplementary information

Supplementary Materials

## Data Availability

The annotated genomic sequences determined for this work have been deposited in the Genbank/EMBL/DDBJ database under the following accession numbers: M. vitis: MG807319, M. australiensis: MG807320, M. maliensis: MK978772, Z. vitis: MG807318, mvtv: MG807316 and matv, MG807317. The mass spectrometry proteomics data have been deposited to the ProteomeXchange Consortium via the PRIDE partner repository with the dataset identifier PXD009037 [75].
