# Supplementary material for: Exploration of the propagation of transpovirons within Mimiviridae reveals a unique example of commensalism in the viral world
Source: ISME J. 2019 Dec 10;14(3):727–39. doi: 10.1038/s41396-019-0565-y (PMC7031253; doi:10.1038/s41396-019-0565-y)
Supplement: Supplementary file 1 — Supplementary Materials [file 41396_2019_565_MOESM1_ESM.docx]

**Supplementary Material**


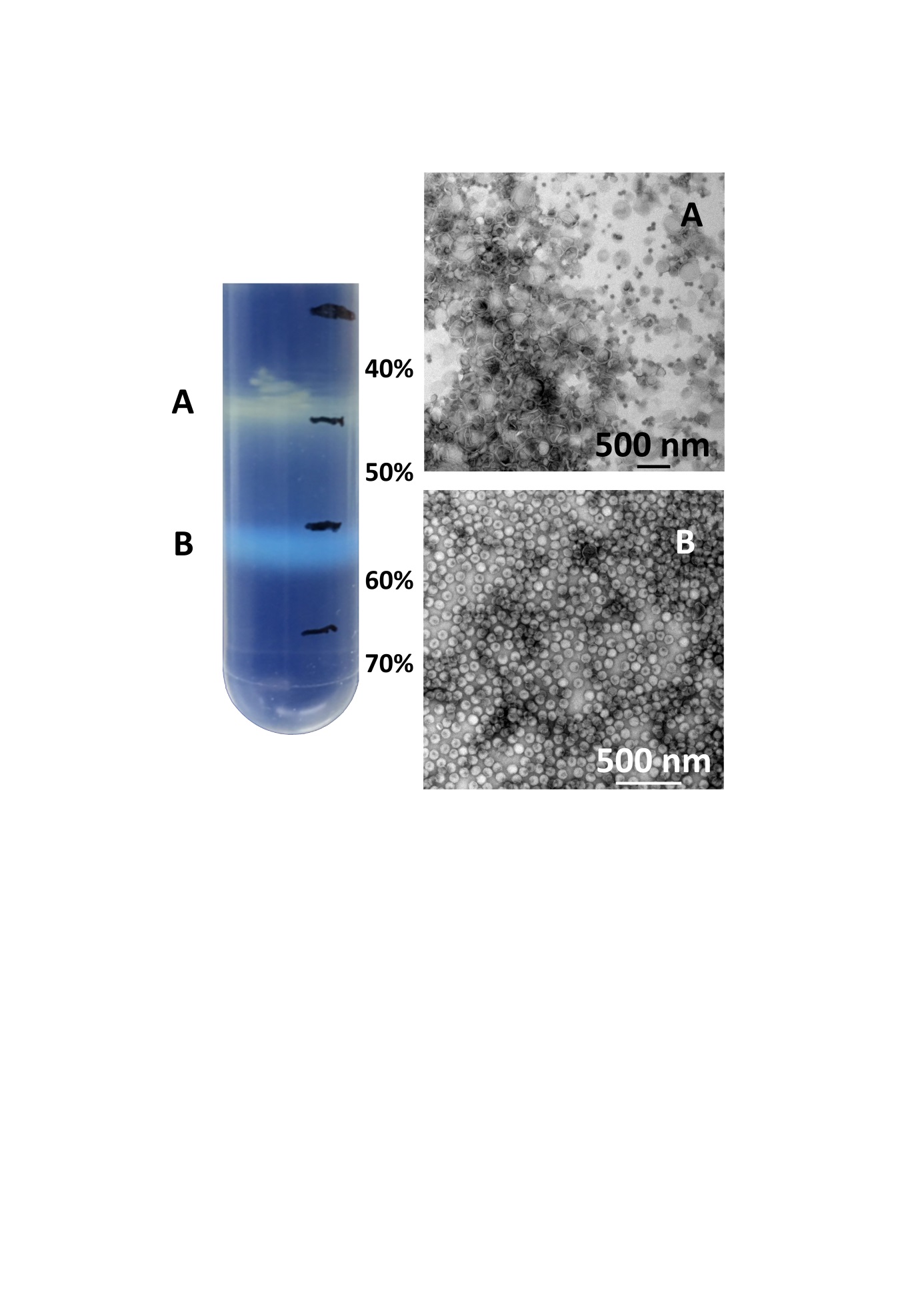


**Figure S1: Virophage purification on sucrose gradient and negative staining images of the recovered disk.** Negative staining confirms that the white disk corresponds to debris (A) and the blue disk to the purified virophage (B).


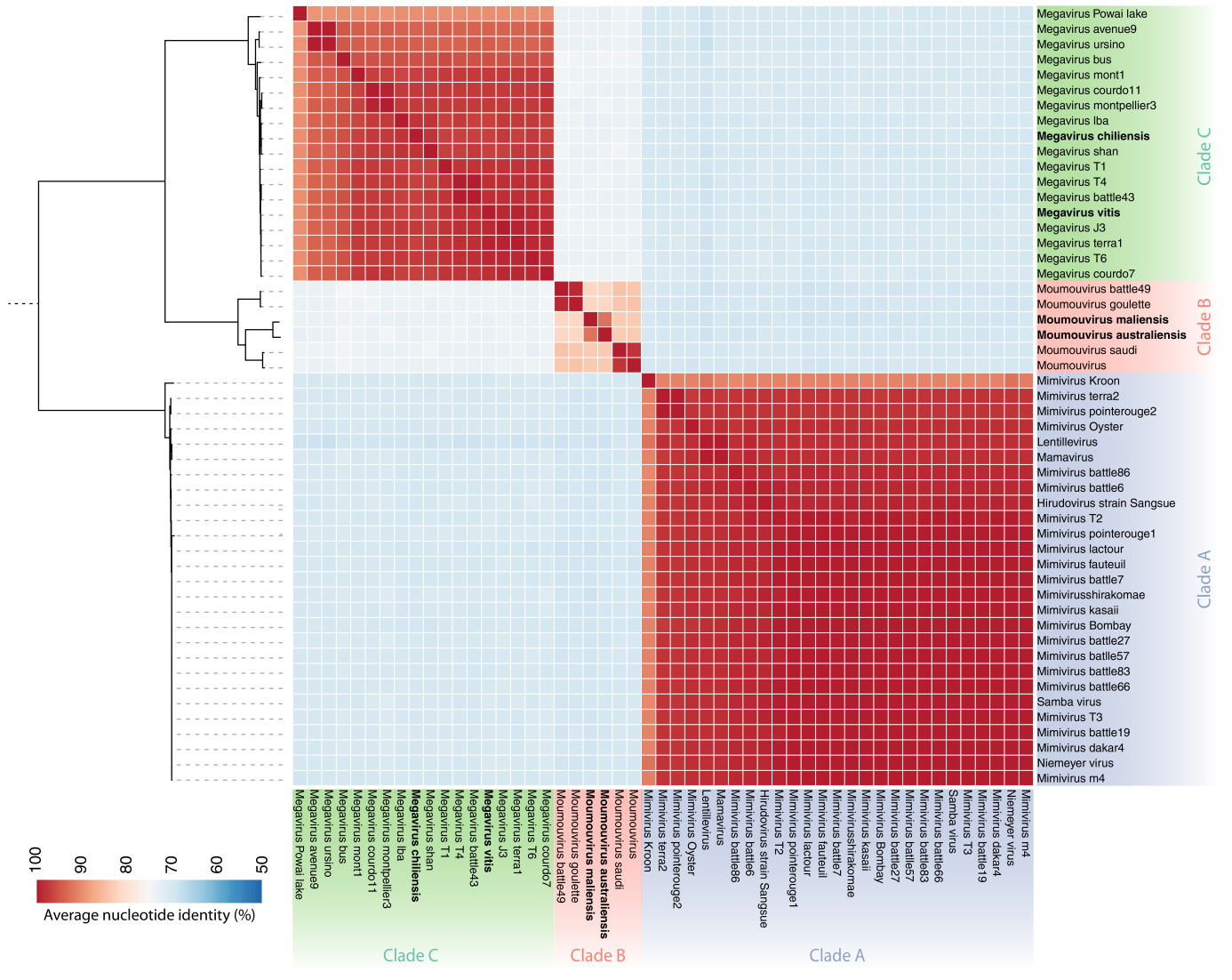


**Figure S2: Genomic sequence conservation and phylogeny of the mimiviruses.**Phylogenetic tree (on the left) was computed from the concatenation of shared orthologous genes peptide sequences using PhyML (1) with the VT model. The heatmap shows the average nucleotide sequence identity between the mimiviruses fully sequenced genomes estimated using the OrthoANIu tool (2).

**
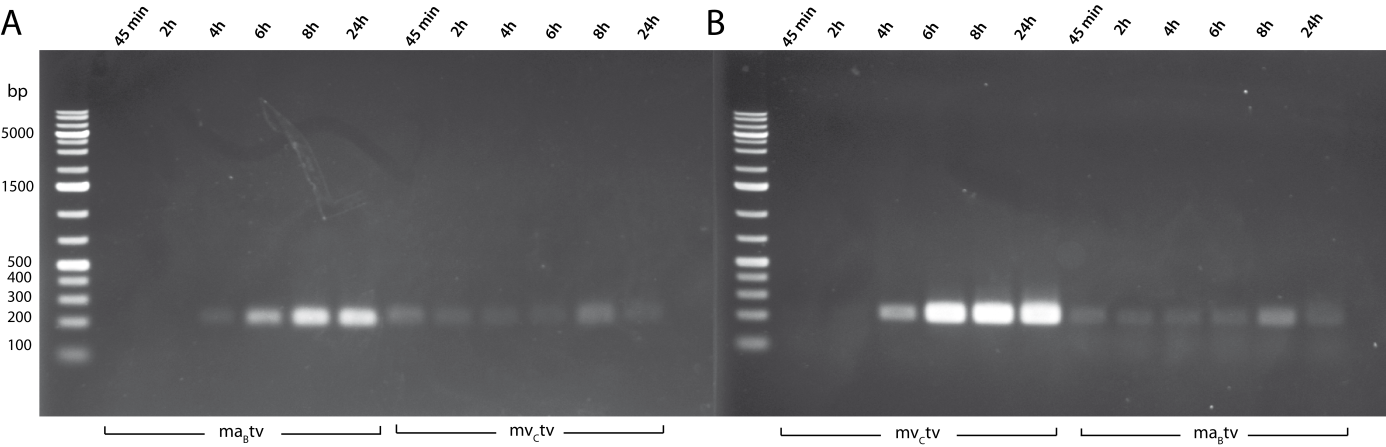
**

**Figure S3: Dominance effect:** PCR specific of each transpoviron were performed using matv and mvtv specific primers *A. castellanii* cell lysates along the infectious cycle of (A) M_B_. australiensis/matv co-infected with a virophage carrying mvtv; (B) M_C_. vitis/mvtv co-infected with a virophage carrying matv. The transpoviron brought is in a large excess at the beginning of the infection (can be detected in the very early time points). The host virus is clearly replicating the transpoviron it carries after 2h pi, while the one brought in by the virophage does not appear to be replicated.

**
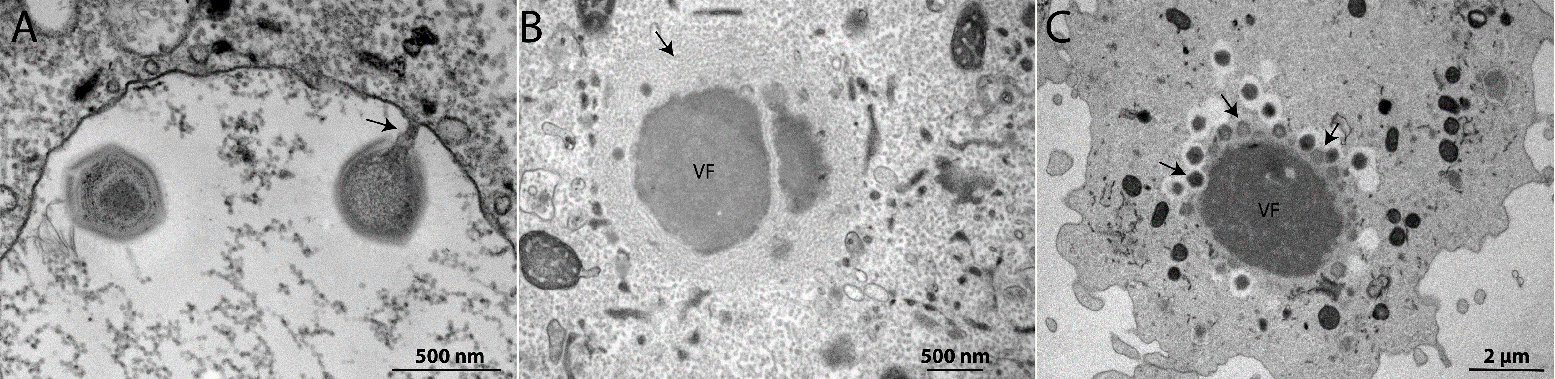
**

**Figure S4: Ultrathin-section TEM imaging of M_C._ vitis-infected Acanthamoeba cells.** (A) At 1 - 2h pi, the giant virus particles are internalized in the vacuoles and the internal membrane of the virus fused with the vacuole membrane (black arrow). (B) At 4h pi viral factories develop in the cytoplasm. A black arrow points to the mesh of fibers that forms, excluding all organelles. (C) Mature viral factories with neo-synthetized virions budding (black arrows) at their periphery.





**Figure S5: TEM comparison of viral factories of M_C_. vitis alone (A-B) with Z. vitis (C-D) or Moumouvirus_B_ australiensis (E-F) co-infected by Z. vitis.** (A-C) Early VF of M_C_. vitis and M_C_. vitis/Z. vitis coinfection prior giant virus virions production. (C) Holes are clearly visible in the VF when Z. vitis is infecting M. vitis and some virophages can be seen (inset) at the periphery of the VF. (B-D) Late VFs. M_C_. vitis virions are clearly visible at the periphery of the VFs and Z. vitis particles can be seen in some holes (inset) (D). (E-F) Late M_B_. australiensis VFs producing giant virus particles and virophages at two different poles of the VF.


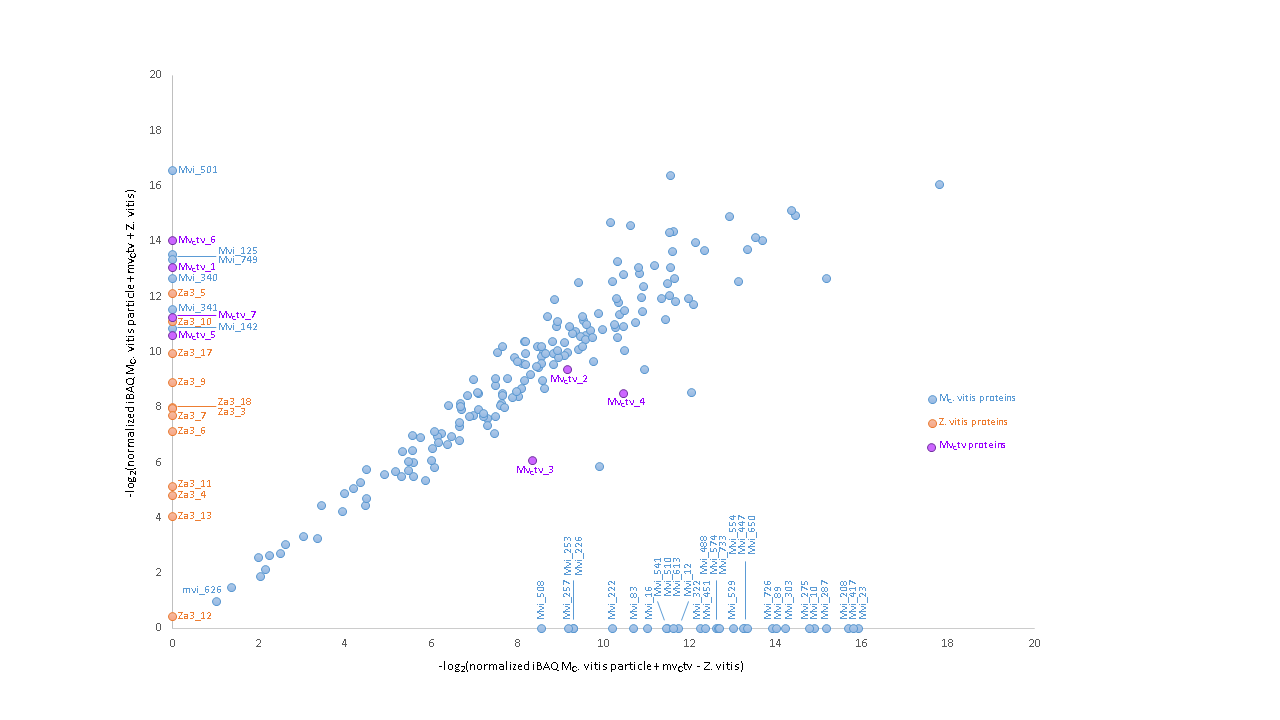


**Figure S6: Proteome comparison of Mv_C_. Vitis purified capsids** resulting from infection of Acanthamoeba cells in the presence or absence of Z. vitis. For the latter, the virophage capsids were not separated from the ones of the giant virus. The abundances of M_C_. vitis (blue), Z. vitis (orange) and mvtv (purple) proteins were compared based on their extracted iBAQ values normalized on the most abundant M_C_. vitis protein (Mvi_626). Higher values correspond to lower protein abundances.


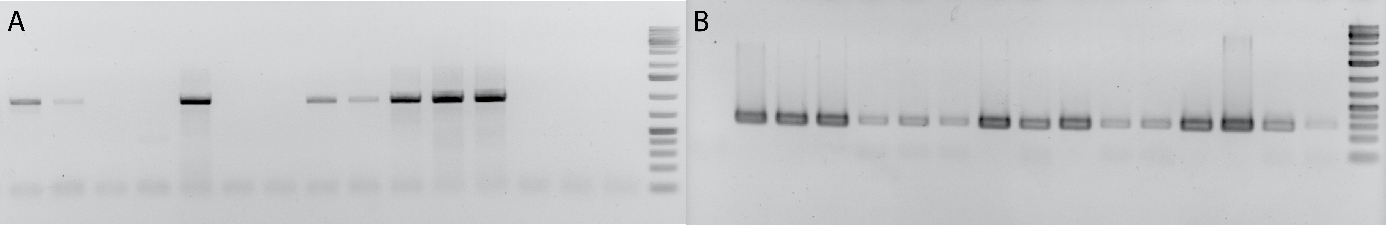


**Figure S7: Agarose gel of PCR performed on various clones of a population of A)** M_C_. chilensis co-infected with a virophage carrying mvtv, using mvtv specific primers and B) M_B_. maliensis co-infected with a virophage carrying matv, using matv specific primers. The two populations of giant viruses correspond to a mixture of transpoviron positive and transpoviron negative particles.

**
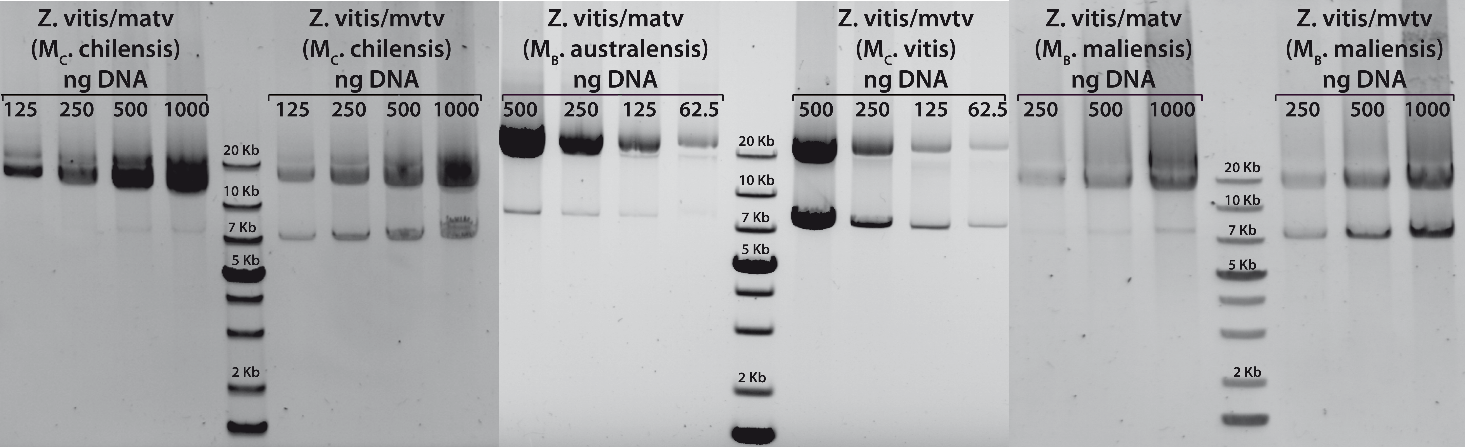
**

**Figure S8: Agarose gel of DNA extracted from purified virophages.** Serial dilutions of DNA extracted from purified virophages associated with matv or mvtv and produced with empty M_C_. chilensis (left gel), M_B_. maliensis (right gel) and M_B_. australiensis/matv or M_C_. vitis/mvtv (middle gel). The mvtv transpoviron is clearly more efficiently associated with the virophage particles than matv, whether produced with M_C_. vitis, M_C_. chilensis or M_B_. australiensis host viruses.


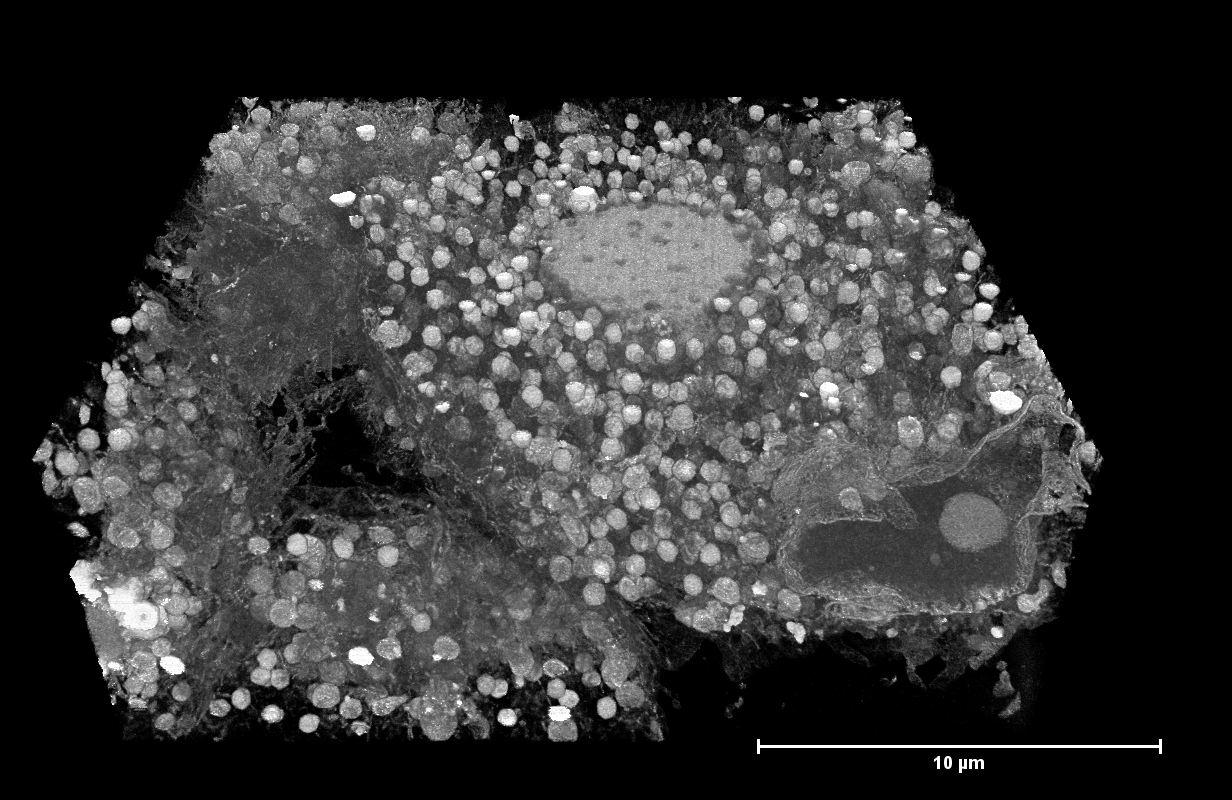


**Supplementary Movie caption: 3D reconstruction of an Acanthamoeba cell co-infected by M_C_. vitis and Z. vitis virophage after 12h pi.** 30 nm slices were acquired on a Scanning Electron Microscope FEI Teneo VS in « Serial Block-Face » mode (263 images 10nmx10nmx10nm and 3 energies deconvolution) and analysed using the VolumeScope™ software. The punctured VF (upper middle) surrounded by maturing M_C_. vitis virions as well as the cell nucleus (bottom right) are easily recognisable. The video can be found under the following link: <https://mycore.core-cloud.net/index.php/s/juqBi1cvPfNF2UX>

**Table S1: Transpoviron proteins identified in the proteomes of host viruses and virophages**

| **Transpoviron proteins** | **Z. vitis purified from M_C_. vitis (+mvtv)** | **Z. vitis purified from M_C_. chilensis (+mvtv)** | **Z. vitis purified from M_B_. australiensis (+matv)** | **Z. vitis purified from M_C_. chilensis (+matv)** | **Z. vitis purified from M_C_. chilensis (no tv)** | **M_C_. vitis (+mvtv)** | **M_C_. vitis infected by Z. vitis (+mv_C_tv)** |
| --- | --- | --- | --- | --- | --- | --- | --- |
| **Mvtv_7/Matv_7** | + | + | + | + | *ND* | *ND* | + |
| **Mvtv_6/Matv_6** | + | + | + | + | *ND* | *ND* | + |
| **Mvtv_1/Matv_8** | + | + | *ND* | *ND* | *ND* | *ND* | + |
| **Mvtv_2/Matv_2** | + | + | + | *ND* | *ND* | + | + |
| **Mvtv_3/Matv_3** | *ND* | *ND* | *ND* | *ND* | *ND* | + | + |
| **Mvtv_4** | + | + | *ND* | *ND* | *ND* | + | + |
| **Mvtv_5/Matv_5** | + | + | *ND* | *ND* | *ND* | *ND* | + |
| *ND: Not Detected* |  |  |  |  |  |  |  |

**Supplementary References**

1. Guindon S, Dufayard J-F, Lefort V, Anisimova M, Hordijk W, Gascuel O. New algorithms and methods to estimate maximum-likelihood phylogenies: 141 assessing the performance of PhyML 3.0. Syst Biol. 2010 May;59(3):307–21.

2. Yoon S-H, Ha S-M, Lim J, Kwon S, Chun J. A large-scale evaluation of algorithms to calculate average nucleotide identity. Antonie Van Leeuwenhoek. 143 2017 Oct;110(10):1281–6.
